# Veterinary News

**Published:** 1894-12

**Authors:** 


### VETERINARY NEWS.

*The Dietetic and Hygienic Gazette*, a journal devoted to physiological medicine, published in New York, contains in its November number a modified milk bill, together with an article by Dr. Leonard Pearson, entitled "Thorough Milk Control Benefits Dairymen," and one entitled "Much Needed Milk Legislation," by Dr. W. H. Ford, of the Philadelphia Board of Health, all of which is well worth reading by those interested in these subjects.

The consideration of retaliatory measures in the Department of Agriculture against those countries that have placed embargoes upon our live-stock and dressed meat products, is stated to have received some consideration at the hands of those who officially represent our country in this direction, and it may result in the inspection of all foods and beverages imported from Germany and Denmark. It is to be hoped that there will be no necessity of resorting to any such selfish measures, and that those governments, who have declined to receive our products, the healthiest and best in the world, will see their error and remove said restrictions.

The State Association, organized at Montgomery, Alabama, on November 13th, after the adoption of the constitution and by-laws, selected the following officers: President, C. A. Cary, Auburn, Ala.; Secretary and Treasurer, Dr. George Joly, Montgomery, Ala.

Missouri has just joined the ranks with a new State organization, her initial meeting bringing together some twenty-five or thirty active members of the profession in that State.

The Central Society of Veterinary Medicine of France in its celebration of its fiftieth year of existence stands hardly without a parallel in the good work it has done for Veterinary Science the world over. We sincerely wish and trust that its future may be as full of good work as its past has recorded. It would have been fitting for the United States Association to have delegated one of its representatives to have joined with them in this anniversary occasion.

Not less than four bills looking to the control and eradication of tuberculosis have been offered at the present session of the Vermont State Legislature. It is hoped that some single measure may be the outcome of this energetic movement on this subject, and that the many bills will not end in the loss of all.

Sir Charles Tucker, Commissioner of Canada, has been appealing to the people of Great Britain to remove the restrictive measures that have been recently imposed against Canadian cattle on the grounds of contagious pleuro-pneumonia. He made the emphatic statement that they had not proven that Canada had ever had a single case of contagious pleuro-pneumonia. The prospects for the lifting of the embargoes are not very promising.

A cablegram announces the probability of Germany appointing Dr. Frank S. Billings, recently of Nebraska Pathological Laboratory, as the one to whom will be referred for decision the gravity of the dangers that Germany claims arise from the importation of American cattle in the direction of Texas fever. This rather bears the impress of a diplomatic move rather than a determination there to settle this question upon an actual basis as to any dangers that may arise in this direction.

Alabama is about to organize a State Association, and as she possesses one of the brightest and most enthusiastic workers of the United States Veterinary Medical Association, we are very sure that it will be a welcome addition to the galaxy of Associations in this country.

Recent investigations on the subject have suggested that blacksmiths should now, in these days of endless electrical wires, place the nails as far distant from the sensitive laminæ as possible, in order to give to the horn its full power as a non-conductor, and that horse owners would do well to keep the hair at the fetlocks cut short, for when it is long and becomes wet it proves a strong conductor in the event of the horse stepping on a live wire.

It is to be hoped that the next session of the Ohio Legislature will not close its session until many of the excellent features of their Act to regulate the practice of Veterinary Medicine and Surgery are restored as they were in the original bill.

There is one great danger that the profession should not fail to measure at its full value, and that is in the establishment of a Veterinary Board of Examiners, the evil consequence that may result for many years in having the bill so drawn that unscrupulous and improperly qualified men may have the seal of approval of their States by virtue of these acts, and thus given a status in the community which they neither deserve nor are likely to use for anything but unscrupulous purposes, and thus reflect great discredit on the profession under the protection of the law. Better had the profession wait until they are stronger rather than accept such legislation at this time.

The Minister of the Interior of Denmark has just placed an embargo against the importation of American live cattle and fresh meat.

The many friends of Dr. S. E. Weber will regret very much to learn of the serious relapse which has befallen him and compelled his retirement again from practice to the confinement of the hospital.

In a recent shipment of live cattle from Baltimore to Liverpool, out of a total of nearly 900 head, a loss of but two occurred en voyage.

The enthusiastic determination of the veterinarians of Iowa and vicinity that the meeting of 1895 of the U. S. V. M. A. shall be held at Des Moines, Iowa, is very much to be commended, and it will make it necessary for the other cities which are bidding for the place of meeting to be up and doing or they will be outrun in the race. Letters are pouring in upon the various members of the Comitia Minora from all parts of Iowa and many of the adjoining States, all of them bespeaking for the meeting, if held in Iowa, one of the most successful in the history of the Association. It is such action upon the part of the veterinarians of our country that bodes a great deal of good for the Association and that tends to give it that national importance which it should command. It makes one almost feel convinced at this writing that Iowa is the place of meeting for '95. What say the other cities?

Philadelphia has added to its new industries an equine meat factory, where horse meat is prepared for foreign markets, where such products are allowed to be sold on the public stalls.

Bulletin, No. 7, of the Bureau of Animal Industry, has just been issued, on Investigations Concerning Bovine Tuberculosis, with a special reference to diagnosis and prevention, as conducted in the Bureau of Animal Industry. This bulletin comes at an opportune time, when this subject is receiving so much serious consideration at the hands of the people at large, and when so much prospective legislation is being considered looking toward a better control and eradication of this disease.

Bulletin, No. 7, is one of the most carefully prepared statistical and scientific reviews of the question of bovine tuberculosis, and it is to be hoped that it will be read and thoroughly considered by every scientific veterinarian, for in addition to shedding a great deal of new light upon the subject, it will be a very great help in also considering this subject from a public point of view in the preparation of future laws for the control and eradication of this serious menace to public health and prosperity.

There is an awakening of public sentiment in intelligent communities of the need of rigid quarantine measures and regulations, that bodes much good for the better control and limiting of the dire results following outbreaks of contagious and infectious diseases. Much of this can be accredited to the earnest and intelligent support of the daily press.

The Alabama State Veterinary Medical Association was organized on November 13th, and while they have but about ten graduates in the State, this promises to be one of the most aggressive State organizations in the list which we have.

Dr. Jas. A. Waugh, late Army Veterinarian, has just recovered from a dangerous attack of typhoid fever, which removed him from professional work for nearly three months.

Prof. W. L. Williams, now holding the chair of Veterinary Science in the Montana Agricultural College and Experiment Station, will shortly issue a bulletin on topics of general interest.

A joint meeting of the Master Horseshoers Union and the Local Journeymen's Union, of Philadelphia, held in Industrial Hall, November 30th, was addressed by Dr. W. Horace Hoskins, President of the U. S. V. M. A., and Dr. D. B. Fitzpatrick, of Philadelphia, in support of the advantages to be derived by the establishment of a school of Farriery, on behalf of the apprentices to the shoeing art. A movement which promises to be extended by branches of the above associations in many of the large cities of this country.

Secretary Morton, in his report for the Department of Agriculture, draws attention to the point, that those who are engaged in the shipment of dressed beef and other meat products to those countries which require an inspection of said meats before shipment, that the cost of said inspection should no longer be paid by the National Government, but should be sustained by those who are reaping the benefit of said inspection. We heartily agree with him on this point.

